# NeDSeM: Neutrosophy Domain-Based Segmentation Method for Malignant Melanoma Images

**DOI:** 10.3390/e24060783

**Published:** 2022-06-02

**Authors:** Xiaofei Bian, Haiwei Pan, Kejia Zhang, Chunling Chen, Peng Liu, Kun Shi

**Affiliations:** Department of Computer Science and Technology, Harbin Engineering University, Harbin 150001, China; xiaofeibian@hrbeu.edu.cn (X.B.); kejiazhang@hrbeu.edu.cn (K.Z.); ccl_00@hrbeu.edu.cn (C.C.); s314067039@hrbeu.edu.cn (P.L.); shikun@hrbeu.edu.cn (K.S.)

**Keywords:** malignant melanoma image, image segmentation, morphology, neutrosophic set, neutrosophic entropy, HGMM

## Abstract

Skin lesion segmentation is the first and indispensable step of malignant melanoma recognition and diagnosis. At present, most of the existing skin lesions segmentation techniques often used traditional methods like optimum thresholding, etc., and deep learning methods like U-net, etc. However, the edges of skin lesions in malignant melanoma images are gradually changed in color, and this change is nonlinear. The existing methods can not effectively distinguish banded edges between lesion areas and healthy skin areas well. Aiming at the uncertainty and fuzziness of banded edges, the neutrosophic set theory is used in this paper which is better than fuzzy theory to deal with banded edge segmentation. Therefore, we proposed a neutrosophy domain-based segmentation method that contains six steps. Firstly, an image is converted into three channels and the pixel matrix of each channel is obtained. Secondly, the pixel matrixes are converted into Neutrosophic Set domain by using the neutrosophic set conversion method to express the uncertainty and fuzziness of banded edges of malignant melanoma images. Thirdly, a new Neutrosophic Entropy model is proposed to combine the three memberships according to some rules by using the transformations in the neutrosophic space to comprehensively express three memberships and highlight the banded edges of the images. Fourthly, the feature augment method is established by the difference of three components. Fifthly, the dilation is used on the neutrosophic entropy matrixes to fill in the noise region. Finally, the image that is represented by transformed matrix is segmented by the Hierarchical Gaussian Mixture Model clustering method to obtain the banded edge of the image. Qualitative and quantitative experiments are performed on malignant melanoma image dataset to evaluate the performance of the NeDSeM method. Compared with some state-of-the-art methods, our method has achieved good results in terms of performance and accuracy.

## 1. Introduction

Malignant melanoma is a dangerous skin cancer, with a substantial death rate [1]. Skin lesion segmentation is the first and indispensable step in the the identification and diagnosis of malignant melanoma [2]. Due to the features shown in skin lesions and the skin itself among different people are diverse, the segmentation work is complicated. Meanwhile, the edges of malignant melanoma are irregular or have notches, etc., and the malignant melanoma does not have a smooth round or oval outline like ordinary nevus. The definition of skin lesion borders is also a difficulty [3,4,5]. Malignant melanoma can occur in any part of the body. It may occur on the skin surface of the abdomen and lower limb skin of the body. It can also appear in areas that do not heal easily, such as the lips, eyes, toenails, and on the surface of cavities such as the nose [6]. Experienced specialists are required when the malignant tumor resections are performed. Meanwhile, if the surgical injury is too large, the injury will not heal easily. If the wound is small, the skin lesion area may not be completely removed, and the patient may get sick again. Therefore, manual image segmentation is a very time-consuming job. It is necessary to accurately segment the skin lesion region, and to improve diagnostic accuracy and effectiveness further.

At present, people have done a lot of research on medical image segmentation and developed many different segmentation methods. The initial skin lesion segmentation method is generally in line with detection method of the edge, region growing, and optimum thresholding. Zhou et al. introduced a Gradient Vector Flow (GVF) algorithm based on mean shift which can correctly drive internal/external energy. The normal GVF cost function is added to the operation of mean shift by using this model. The borders of skin lesions in images can be accurately determined by the proposed method [7]. The Expectation Maximization (EM) facial skin color segmentation algorithm is proposed. The algorithm uses color space knowledge and different light conditions to correct and segment the front and side facial color images. It has a better effect on segmenting facial images by the presented EM algorithm [8]. Ehsan used bilateral filtering to form a fuzzy C-means (FCM) algorithm for segmenting an image. This filter is used to reduce noise and make a soft picture. It could avoid the over-segmentation of images through the method which is on the basis of the pixels [9]. Xu and Mandal proposed the method to realize the segmentation of coarse epidermis based on global thresholding and shape analysis. Moreover, the line segments perpendicular to the central axis of the initially segmented epidermis mask are applied to measure the thickness of epidermis. The K-means algorithm is used to perform secondary precision segmentation on these coarse segmentation results to improve the performance [10]. Shi X et al. proposed a Hierarchical Gaussian Mixture Model (HGMM), the model can segment the high-resolution remote sensing image well. The weighted sums of elements are used to define these components to accurately simulate the complex distributions of pixel intensities in object areas. In order to simulate the pixel intensity distribution in the local area of the object area, Gaussian distribution is applied to determine the element of the component. In the light of the Bayesian theorem, the HGMM and the prior distributions of parameters are combined to establish the segmentation model. [11]. Wang Y et al. proposed a hierarchical Gaussian mixture model segmentation algorithm on the basis of Markov random field (MRF). The hierarchical processing of Gaussian mixture model is applied for replacing the traditional Gaussian mixture model distribution, the MRF is constructed for image pixels, and the prior probability of the hierarchical Gaussian mixture model is updated [12]. However, a lot of manual participation are needed and a lot of hyperparameters need to be adjusted in order to obtain better segmentation results of these traditional methods.

In recent years, deep learning technology has developed rapidly, more and more models are used in medical images, especially for skin lesion area segmentation. The literature built a Fully Convolution Residual Network (FCRN) in the line with the Fully Convolutional Network (FCN). The feature extraction capability for skin lesion segmentation is improved by combining multi-scale context information [13]. Oktay et al. proposed adding Attention Gate (AG) to U-net skipping connections to improve prediction accuracy and sensitivity in a pancreas segmentation protocol [14]. A novel loss function is proposed to make the task of segmentation better, and it is on the basis of Jaccard distance. A U-shaped network (U-net) is built in the literature, and it is now widely used to segment skin lesions [15]. A dense deconvolutional network and U-net combined for the automatic segmentation of skin lesion is introduced [16,17], where multiple dense blocks are stacked together to improve the representativeness of the model [18,19]. Deshpande N M et al. explored an efficient and hybrid segmentation that proposes a more efficient and effective system for leukemia diagnosis. A very popular publicly available database, the acute lymphoblastic leukemia image database (ALL-IDB), is used in this research. First, the images are pre-processed and segmentation is done using Multilevel thresholding with Otsu and Kapur methods. To further optimize the segmentation performance, the Learning enthusiasm-based teaching-learning-based optimization (LebTLBO) algorithm is employed [20]. Agrawal R et al. proposed to employ two modified U-Net architectures to segment the demarcation line/ridge and vessel automatically, namely, squeeze and excitation U-Net (SE U-Net) and U-Net with attention gates (AG U-Net). In basic U-Net, each channel of feature maps is weighted equally, whereas, in SE U-Net, the weights of each channel are adjusted adaptively by adding parameters to it. The AG U-Net selects only salient features of an image and prunes the irrelevant features by applying a weighted feature map function for each object and paying attention to a particular object in an image [21]. The novelty of the literature lies in detailed analysis and discussion of U-Net++ results and implementation of U-Net++ in lung segmentation using X-ray. A thorough comparison of U-Net++ with three other benchmark segmentation architectures and segmentation in diagnosing TB or other pulmonary lung diseases is also made in the literature [22]. A Full convolutional Residual Network (FcRN) for end-to-end training is proposed, and it obtains a better result of segmentation [23]. U-Net and Deeplabv3 are combined to built DeepLabv3+, a decoder module to recover the boundaries of the object is added as an extension of Deeplabv3. Recently, attention-based networks have been diffusely used in various problems of computer vision [24]. Mu et al. proposed channel context and dual-domain attention based U-Net (CDU-Net) for segmentation of skin lesion attributes. The channel context feature fusion module (CCM) is used to further extract context information. dualdomain attention (DA) modules are further combined to improve the accuracy of segmentation result [25]. Jignesh G. et al. proposed a new convolutional neural network-based approach for skin lesion segmentation. Meanwhile, a novel multi-scale feature extraction module is proposed for extracting more discriminative features for dealing with the challenges related to complex skin lesions; this module is embedded in the U-Net, replacing the convolutional layers in the standard architecture. Further, two different attention mechanisms refine the feature extracted by the encoder and the post-upsampled features [26]. Nida N et al. proposed a deep learning method for automatic detection and segmentation of melanoma regions within the dermoscopic images for precise melanoma segmentation. The proposed method generates bounding boxes around multiple regions to precisely detect the affected regions using RetinaNet. Further, conditional random field (CRF) is applied to the detected regions for segmentation of the melanoma lesion [27]. However, many segmentation methods using convolutional neural networks often fail to extract lesion boundaries accurately. Meanwhile, the edges of the lesions in medical images are fuzzy and constantly oscillating. However, deep learning methods do not preserve edges very well. Meanwhile, deep learning methods always require a high cost of medical image collection and labeling, it is difficult to collect enough and balanced training samples for medical image segmentation.

Neutrosophic Set (NS) theory provides a new method for solving uncertainty problems. NS method has been used in image processing and has achieved good results [28]. In general, the perception of the fuzzy-based approaches is generalized by the neutrosophic set. It includes the fuzzy set and intuitionistic fuzzy set [29]. In the NS domain, Guo and Cheng introduced fuzzy c-means clustering, and the indeterminacy of the image is calculated by the entropy. The research put forward an image segmentation model, the model combines NS theory with level set theory. The uncertainty of the image is reduced by using a filter which is newly defined. The boundary of the object is automatically extracted by using the level set algorithm [30]. Cheng and Guo converted the image in NS domain, and segmented the image through the thresholding-based segmentation method. The three NS memberships, that is True (T), Indeterminacy (I), and False (F) are generated. Next, the indetermination is estimated by calculating the entropy in NS. The uncertainty of the set is reduced by applying the mean operation [31,32]. Sert and Alkan proposed a Chan Vese segmentation approach on the basis of NS, and it is used for detecting the edge [33]. Zhang et al. combined the neutrosophy theory with image segmentation, the image is mapped in the NS domain. The image is segmented by using a watershed method [34]. Shan et al. proposed an neutrosophic L-means (NLM) for segmenting images. The method is a clustering procedure on the basis of NS [35]. However, the three memberships (T, I, F) in the neutrosophic set are independent of each other. In order to simulate human decision-making in the process of image segmentation, a single-valued Neutrosophic Entropy (NE) is constructed through T, I, and F to effectively represent the fuzzy boundary features of skin lesion area, healthy skin area and banded edge area in the image. The proposed NeDSeM method further improves the accuracy of the uncertainty of things reflected by the neutrosophic entropy. In this way, it can improve the description of the fuzzy features of the banded edge of the skin lesion area and assist the doctor in effectively resecting the skin lesion area. All knowledge is obtained from the image by unsupervised learning without too much reliance on human experience, and the result is more objective.

Although the above methods have achieved better results, many methods are difficult to transfer to the real world. Compared with the fixed human body structure in other medical images, the distribution of skin lesions in skin diseases often does not have inherent laws. For the segmentation of malignant melanoma, to the best of our knowledge, the representation of images in the existing methods does not conform to the medical diagnosis rules, and their segmentation results retain less edge information. Neutrosophy domain-based segmentation method (NeDSeM) is proposed in this paper according to the background of malignant melanoma image segmentation. Aim of our work is automatically confirm the resection range of skin lesions and effectively segment the edges of skin lesions in malignant melanoma images. It can assist doctors to perform precise skin lesions resection. The proposed method is evaluated on real dataset. Experimental results show that the segmentation accuracy of our method exceeds baseline methods. Overall, the main contributions of our work are listed below.

- In this paper, a segmentation method integrated with morphology and neutrosophy is proposed to segment the banded edges in malignant melanoma images well. In this method, the pixel matrixes of images are optimized at multiple levels. The method in this paper can quickly and accurately obtain better segmentation effect by clustering the final optimized pixel matrixes.
- Due to the banded edges between the skin lesion areas and the healthy skin areas in malignant melanoma images have stronger uncertainty and fuzziness. Neutrosophic Set is used to solve this problem that is superior to a fuzzy set. The three memberships in the neutrosophic set can well highlight the banded edges of images. This paper incorporates the closing operation and the gradient of the morphology into the neutrosophic set conversion process to get these memberships.
- A new neutrosophic entropy model is proposed to comprehensively express T, I, F and highlight the banded edges in the images. The neutrosophic entropy is explained from its spatial geometric meaning, and a new concept of the isentropic cylinder is proposed in the perspective of the intuitiveness and the fuzziness.

Section Methods provides an overview of the proposed segmentation method and discusses the detail of the model. Section Experiments presents the performance evaluation of the method for malignant melanoma image segmentation. Section Conclusions concludes the paper and discusses the possible future works.

## 2. Methods

### 2.1. Overview of the Method

**Figure 1:**
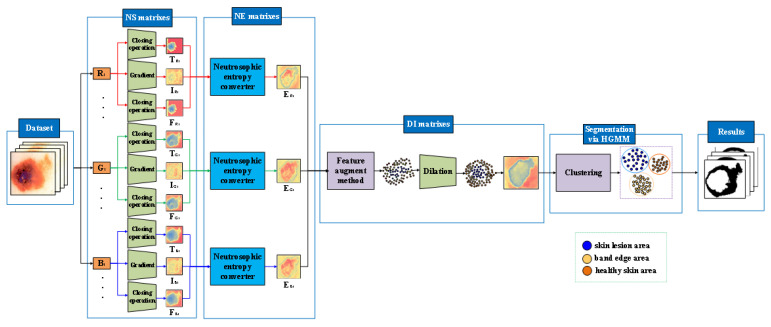
The framework of the NeDSeM method.

In this paper, an automatic segmentation framework for malignant melanoma images is proposed, as shown in Figure 1. Firstly, an image is converted into three channels, that is R, G and B, and the pixel matrix of each channel is obtained. Secondly, the boundaries between the skin lesion areas and the healthy skin areas in malignant melanoma images are fuzzy and uncertain. Therefore, a malignant melanoma image can be subdivided into a skin lesion area, a healthy skin area and a banded edge area between the skin lesion area and the healthy skin area. The three memberships in the neutrosophic set can well highlight the banded edges. NS is more successful than a fuzzy set in solving uncertain situations, especially in the application of image edge detection and segmentation. Therefore, the pixel matrixes are converted from RGB domain into NS domain by using the neutrosophic set conversion method. The NS matrixes are obtained and denoted as (T_R_, I_R_, F_R_), (T_G_, I_G_, F_G_) and (T_B_, I_B_, F_B_). In the process of conversion, due to the closing operation in the morphology can be used to eliminate the holes or the small points in the objects and restore the real pixels of the target images. Therefore, the closing operation is used in the conversion process of T and F. Due to the gradient in the morphology is applied to highlight the edge contours of the target objects, it can represent the degree of uncertainty of T, I and F. Therefore, the gradient is used in the conversion process of I. Thirdly, the colour of the edges between the skin lesion areas and the healthy skin areas in malignant melanoma images is gradual change. However, the three memberships in the neutrosophic set are independent of each other. Therefore, a new neutrosophic entropy model is proposed to combine the three memberships according to some rules by using the transformations in the neutrosophic space. It can comprehensively express T, I, F and highlight the banded edges of the objects. It can map the two-dimensional image pixels to the three-dimensional neutrosophic space one by one. The irregular and uneven pixels in the image can be regularized by isentropic cylinder. Meanwhile, it can also represent the pixel distribution characteristics of malignant melanoma dermoscopic images and facilitate the calculation of segmentation model. The neutrosophic entropy matrixes are obtained and denoted as E_R_, E_G_ and E_B_. Fourthly, the edges of the skin lesion areas of malignant melanoma images are irregular and constantly changing. It is difficult to describe the edge features accurately by using only a single component. Therefore, a feature augment method is established by the difference of the three components. It can reduce the complexity of the model and the consumption of computing resources. The effective edge information of malignant melanoma images are retained as much as possible. The neutrosophic entropy matrixes after using the feature augment method are denoted as R_b_. Fifthly, due to the dilation operation can enlarge the binary areas according to the structuring element, the dilation is used on the neutrosophic entropy matrixes and the matrixes after dilation operation (DI matrixes) are obtained. Finally, the image that is represented by transformed matrix is segmented by the Hierarchical Gaussian Mixture Model clustering method to obtain the banded edge of the malignant melanoma image.

### 2.2. Morphology-Based Operations

In this section, the closing operation, the gradient and the dilation in morphology are introduced, respectively. The closing operation and gradient are used in the process of NS conversion. The holes or the small points in the object can be eliminated by using the closing operation in the morphology and the real pixels of the target image can be restored. So the closing operation is used in the conversion process of T and F. The gradient is used to highlight the edge contour of the target object and it can represent the degree of uncertainty, so the gradient is used in the conversion process of I. The dilation operation can enlarge the areas according to the structuring element, so the dilation is used before clustering.

**Figure 2:**
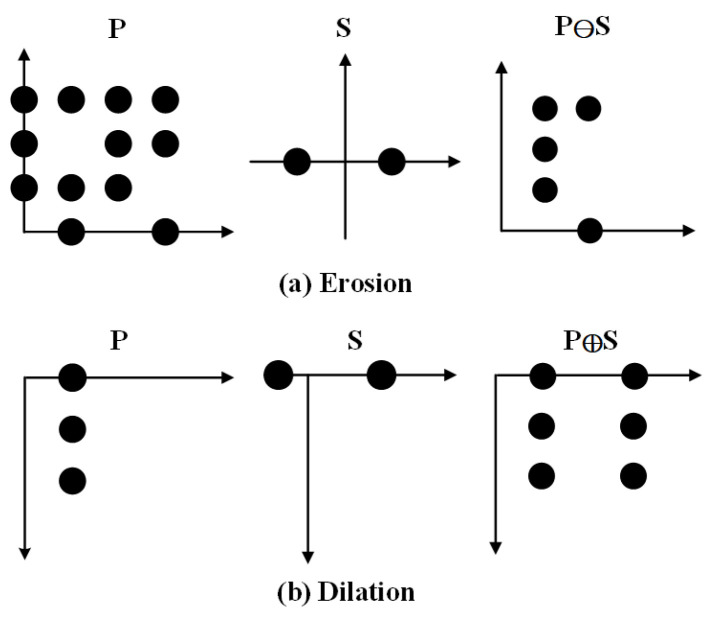
Examples of corrosion and expansion processes.

Firstly, the pixel matrixes of R, G and B in the images are extracted. Morphology is a nonlinear filtering method. The basic idea is to measure and extract corresponding shapes to achieve image analysis and object identification using structural elements with a certain morphology. Morphological operation is formed by the interaction of the object shape set and structural elements. It is not only can suppress noise, but also can detect the real edge greatly [36]. Thus, the use of morphology can not only effectively filter out noise but also retain the original detail information in the image, and it has a better edge detection effect. The morphology contains two basic operations: Erosion and Dilation [37]. Examples of corrosion and expansion processes are shown in Figure 2. For an image, the results of erosion and dilation operation can be obtained.

(1) $$M_{E}=P⊖S=\left\{x,y|S_{\mathrm{xy}}\subseteq P\right\}$$

(2) $$M_{D}=P⊕S=\left\{x,y|S_{\mathrm{xy}}\cap P=\phi \right\}$$

where the erosion and dilation operations are represented by ⊖ and ⊕, respectively. The results of the erosion and dilation are M_E_ and M_D_. The pixel matrix of the image is denoted as P. The structuring element is denoted as S. Compared with structuring element, the smaller areas are removed by erosion operation. On the contrary, the binary regions are enlarged in the light of structuring element by using dilation operation.

Based on the above formulas, the closing operation is obtained as the following:

(3) $$M_{C}=P•S=\left(P⊕S\right)⊖S$$

where the closing operation is represented by •, and it denoted as M_C_. The pixel matrix of the image is denoted as P. The structuring element is denoted as S. The closing operation has two steps, step 1: the erosion operation is applied to filter the binary area; step 2: the dilation operation. The holes to connect the whole binary region are eliminated by the closing operation.

The intensity of pixels in the image can be enhanced through morphological gradient based on the difference between dilation and erosion. It is used to highlight the edge contour of the target object. The mathematical expression is as follows:

(4) $$M_{G}=M_{D}-M_{E}$$

where the morphological gradient is denoted as M_G_. M_D_ and M_E_ represent the results of the dilation and erosion.

### 2.3. The Neutrosophic Set Domain Conversion

The boundaries between the skin lesion areas and the healthy skin areas in malignant melanoma images are fuzzy and uncertain. The three memberships in the neutrosophic set can well highlight the banded edges of the areas. In this section, the pixel matrixes are converted into the form of neutrosophic set by using the neutrosophic set conversion method. In the process of conversion, the closing operation and the gradient in the morphology are used. Then the fuzziness of image edge can be solved better.

Neutrosophic Set (NS) is a useful theory for computing fuzzy situations. In recent years, NS has made a initial attempt in the applications of image processing. NS is more successful than a fuzzy set (FS) in solving uncertain problems, especially in detecting the edge of image and segmenting image [38,39]. All image pixels are subdivided into T, I, and F memberships when the NS theory is applied in image segmentation.

The images are converted to the NS domain, and the images are processed in the NS field [40]. P(i, j) is the pixel matrixes in the original images, where i = 1, …, m, j = 1, …, n. P_C_ is the pixel matrix set of P(i, j) in R, G and B. The conversion formula is the follows:

(5) $$P_{C}=\left\{R\left(i,j\right),G\left(i,j\right),B\left(i,j\right)\right\}$$

(6) $$\;{\hat{p}}^{\prime }=M_{C}\left(\;p^{\prime }\right)$$

(7) $$T\left(i,j\right)\bar{=}\frac{\;{\hat{p}}^{\prime }\left(i,j\right)-{\;{\hat{p}}^{\prime }}_{\min}^{}}{{\;{\hat{p}}^{\prime }}_{\max}^{}-{\;{\hat{p}}^{\prime }}_{\min}^{}}$$

(8) F(i,j)=1−T(i,j)

(9) $$\delta \left(i,j\right)=\mathrm{abs}\left(M_{G}(\;p^{\prime })\right)$$

(10) $$I\left(i,j\right)=\frac{\delta \left(i,j\right)-\delta_{\min}}{\delta_{\max}-\delta_{\min}}$$

where, P′ are the pixel matrixes of any channel in P_C_. P′ are converted into $\;{\hat{p}}^{\prime }$ by the closing operation in morphology, where $\;{\hat{p}}^{\prime }\left(i,j\right)$ is the pixel matrixes of $\;{\hat{p}}^{\prime }$, the maximum and minimum values of $\;{\hat{p}}^{\prime }$ are ${\;{\hat{p}}^{\prime }}_{{}^{{}_{\max}}}^{}$ and ${\;{\hat{p}}^{\prime }}_{{}^{{}_{\min}}}^{}$, respectively. The sum of T and F is 1. The pixel matrixes of δ(i, j) are obtained by the morphological gradient conversion and absolute value calculation for P′. δ_max_ and δ_min_ are the maximum and minimum values of δ, respectively.

In summary, the neutrosophic matrixes of R, G and B are obtained, denoted as (T_R_, I_R_, F_R_), (T_G_, I_G_, F_G_) and (T_B_, I_B_, F_B_).

### 2.4. The Neutrosophic Entropy Domain Conversion

**Figure 3:**
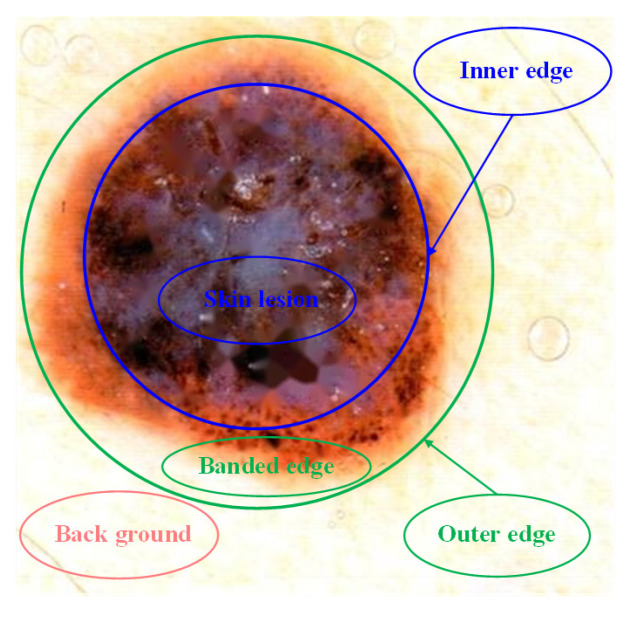
The edge of malignant melanoma image. The malignant melanoma image can be divided into three areas. Outside the outer edge is the background area; Between innter edge and outer edge is the fuzzy edge of the skin lesion, that is, the banded edge; Inside the inner edge is the skin lesion area.

Due to the color of the edge of malignant melanoma image is gradual, it cannot effectively define the boundary between the lesion area and the skin area. As shown in Figure 3, the banded edge is existed between the healthy skin area and the skin lesion area in the malignant melanoma image.

In Figure 3, there is a gradually fuzzy banded edge between the inner edge and the outer edge. When doctors often determine the cutting range of the skin lesion in the banded edge, there is no effective en-bloc resection due to human subjective factors. The colour of the edge between the skin lesion area and the healthy skin area in malignant melanoma image is gradual change. However, the three memberships in the neutrosophic set are independent of each other. Therefore, the neutrosophic entropy model is introduced to combine the three memberships according to some rules by using the transformations in the neutrosophic space. It can comprehensively express T, I, F and highlight the banded edges of the objects. In this section, a new neutrosophic entropy model is proposed from the perspective of the intuitiveness and the fuzziness of image lesion features. The neutrosophic entropy is explained from its spatial geometric meaning, which further improves the accuracy of the uncertainty of things reflected by the neutrosophic entropy. A new concept of the isentropic cylinder is proposed, and a new neutrosophic entropy model is constructed. The existing neutrosophic entropy method can describe the fuzzy decision-making process of doctors during diagnosis, it can help doctors determine the cutting position of the lesions during the operation. However, it only transforms for pixel values and does not consider the distribution of pixels in the dermatoscopic image. Therefore, the concept of isentropic cylinder is proposed. On the two-dimensional plane, the distribution of the same or similar pixels are irregular and uneven, so a new neutrosophic transformation method is proposed to map the points on the two-dimensional image to three-dimensional space of the neutrosophic one by one. It ensures that the original similarity relationship of pixels remains unchanged, so that these similar points fall on a cylinder determined by a fixed axis and radius. The results after image pixel mapping processing can be expressed explicitly, which can represent the pixel distribution characteristics of malignant melanoma dermoscopic images. Meanwhile, it is beneficial to the calculation of segmentation model. (T_R_, I_R_, F_R_), (T_G_, I_G_, F_G_) and (T_B_, I_B_, F_B_) are input into the neutrosophic entropy model for calculation, respectively.

**Figure 4:**
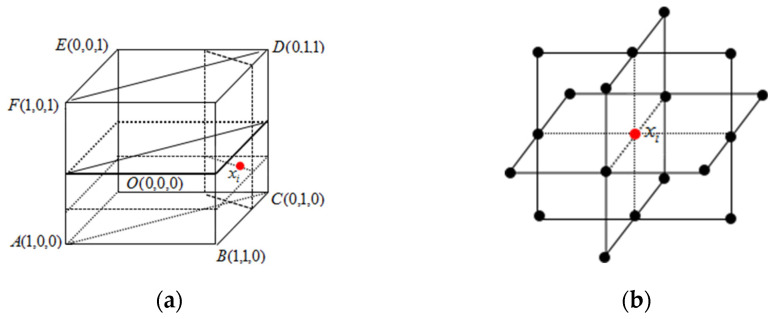
(**a**) The position of point x_i_ in the cube (**b**) The key points around point x_i_.

Now the comparison of entropy between a point x_i_ in the cube and several key points around it is considered, as shown in Figure 4a,b. Ali proposed a single-valued neutrosophic entropy *E* [41,42]:

(11) $$E=\frac{1}{n}\sum_{i=1}^{n}\left(1-\frac{1}{b-a}\int_{a}^{b}\left|t-f||i-i_{c}\right|dx\right)$$

The neutrosophic set contains both the membership information, namely *t*, *f*, and the unknown information, namely *i.* Therefore, the neutrosophic set should be composed of two parts, one is fuzziness, which can be measured by |t − f|, and the other is intuitiveness, which can be measured by |i − i_c_|.

**Figure 5:**
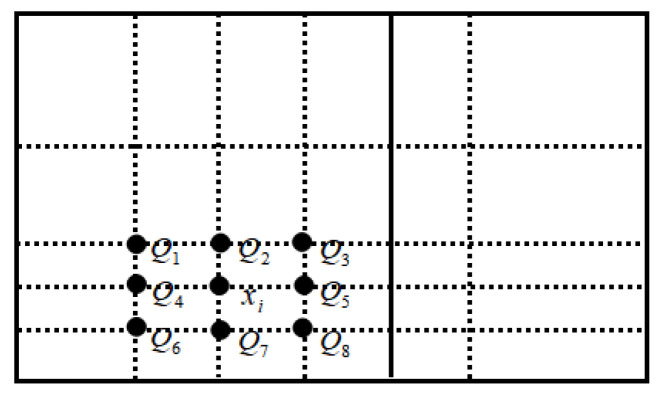
The position relationship between point x_i_ and surrounding key points projected on plane ACDF.

**Figure 6:**
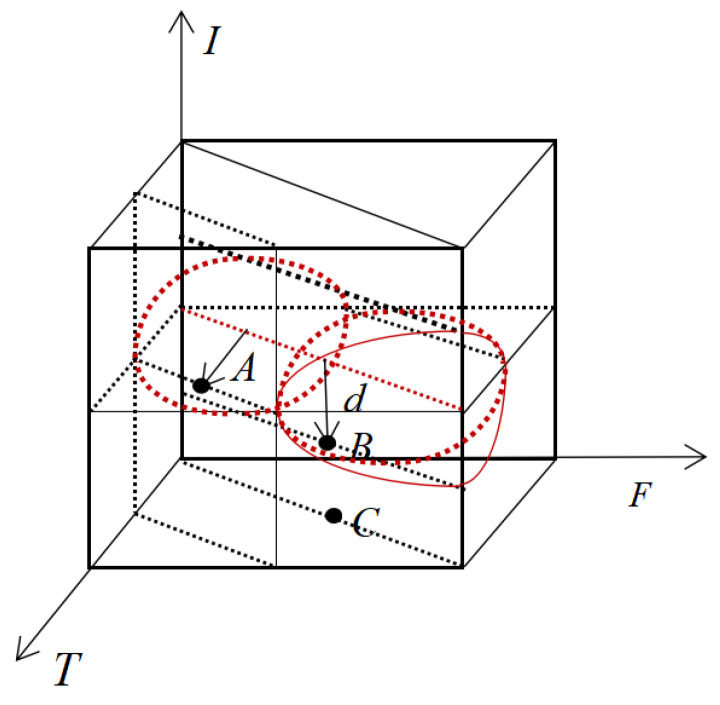
The geometric meaning of single-valued neutrosophic entropy.

For the convenience of comparison, all points are projected on plane ACDF, as shown in Figure 5. Finally, only the entropy between Q_i_ and x*_i_ is compared, and it has E(Q_4_) < E(x_i_) < E(Q_5_), E(Q_7_) < E(x_i_) < E(Q_2_). Because of E(x_i_) < E(Q_2_) and E(Q_2_) < E(Q_3_), E(x_i_) < E(Q_3_) is obtained. The same is true for E(x_i_) > E(Q_6_), that is, E(Q_6_) < E(x_i_) < E(Q_3_). However, it is impossible to compare point x_i_ with point Q_1_ and point Q_8_, because when x_i_ moves to Q_1_ or Q_8_, increase and decrease characteristics of its intuitiveness and fuzziness are opposite. It is impossible to intuitively judge which aspect is more important through geometric figures, so the entropy cannot be compared. To solve this problem, a cylindrical surface is made with the intersection line $\left\{ \begin{array}{l} z=0.5\\ t=f \end{array}\right.$ of plane z = 0.5 and plane EGBO as the axis and d as the radius, as shown in Figure 6. As point A approaches point B, the fuzziness increases and the intuitiveness decreases. We can assume that their interactions cancel each other out, so that the uncertainty information at this point is constant, that is, the entropy is constant, that is, E(A) = E(B), this arc is called an isentropic cylinder.

As can be seen from Figure 6, the points on the isentropic cylinder have the feature of equal entropy, and the entropy has an obvious decreasing trend with the increase of d. Therefore, the neutrosophic entropy corresponding to this point can be measured according to the distance between any point in the cube and the straight line $\left\{ \begin{array}{l} z=0.5\\ t=f \end{array}\right.$. A more reasonable and reliable entropy formula can be established based on this. Theorem 1 can be obtained after the research.

**Theorem** **1.** *Suppose X is a domain, X = {x_1_, x_2_,* *…, x_n_}. It is a non-empty set, and the element of it is denoted as x.* *A = {x_i_, <t_A_(x_i_), i_A_(x_i_), f_A_(x_i_)> x_i_* *∈X}SVNS(X) is called as a Neutrosophic Set (NS) on X. Where T_A_(x), I_A_(x), and F_A_(x) are the membership degree, the hesitancy degree, and the non-membership degree of the element x in X belonging to set A. For “x* *∈X,*

(12) $$T_{A}(x):X\to {]}^{-}{0,1}^{+}[$$

(13) $$I_{A}(x):X\to {]}^{-}{0,1}^{+}[$$

(14) $$F_{A}(x):X\to {]}^{-}{0,1}^{+}[$$

*and* *−0* *≤ T_A_(x) + I_A_(x) + F_A_(x)* *≤ 3+. That is, the three functions are independent of each other. For the convenience of study, it is called* *α =< T, I, F> is a neutrosophic number.*

*Where ]* *^−^0,1^+^[ is the non-standard unit interval relative to the closed interval [0,1]. If*

(15) $$\begin{array}{l} E=\frac{1}{n}\sum_{i=1}^{n}\left\{1-\frac{2\sqrt{3}}{3}\sqrt{\frac{{{(t(x}_{i})-{f(x}_{i}))}^{2}}{2}+{\left(\frac{\left|{I(x}_{i})-I^{c}{(x}_{i})\right|}{2}\right)}^{2}}\right\}\\ =\frac{1}{n}\sum_{i=1}^{n}\left\{1-\frac{2\sqrt{3}}{3}\sqrt{\frac{{{(t(x}_{i})-{f(x}_{i}))}^{2}}{2}+{\left(\frac{|1-{2I(x}_{i})|}{2}\right)}^{2}}\right\} \end{array}$$

*Then E is the entropy of the intuitionistic fuzzy set*.

In summary, the neutrosophic entropy matrixes are obtained, denoted as E_R_, E_G_ and E_B_. The results processed by neutrosophic entropy not only better solve the problem of fuzzy edges of malignant melanoma images, but also more consistent with the decision-making of human. The detailed derivation process and some properties of single-valued neutrosophic entropy can be found in Supplementary Materials File S1.

### 2.5. Segmentation Method

The edges of the skin lesion areas of malignant melanoma images are irregular and constantly changing. Therefore, the difficulty of edge feature extraction is that it is difficult to describe using only a single component accurately. However, much computations are required by using three components for description at the same time. In this paper, the edge feature of the malignant melanoma image is established by the difference among the three components to reduce the complexity of the model and the consumption of computing resources. At the same time, the effective edge information of the malignant melanoma image is retained as much as possible. The influence of the blue channel component on feature extraction is considered. It is necessary to eliminate the area with a higher blue component value in the red component. Therefore, the edge feature of the malignant melanoma image is extracted, denoted as:

(16) $$R_{b}=E_{R}-E_{B}$$

where E_R_ is the neutrosophic entropy conversion result of channel R, and E_B_ is the neutrosophic entropy conversion result of channel B.

**Figure 7:**
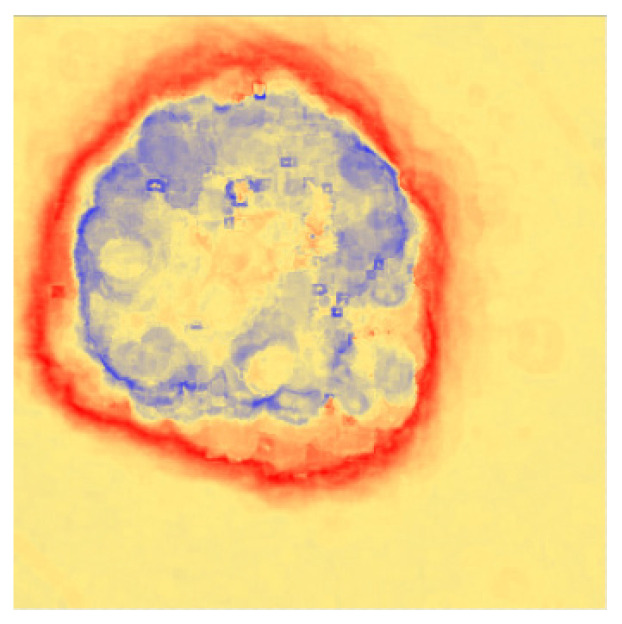
The sample graph of R_b_.

Due to the dilation operation can enlarge the binary areas according to the structuring element, the dilation is used on the neutrosophic entropy matrixes. DI matrixes are obtained by using Formula (2). The processed image is shown in Figure 7.

Finally, the Hierarchical Gaussian Mixture Model clustering method is used to segment the skin lesion areas of malignant melanoma images.

The HGMM algorithm adds hierarchical processing on the basis of traditional Gaussian Mixture Model, and performs K Gaussian mixture modeling again with K Gaussian distributions [12]. That is, the Gaussian mixture model is used to replace the kj*th* Gaussian distribution. Suppose the image P contains N pixels, P = (p_1_, …, p_n_), the following density function is defined to divide the image into K categories.

(17) $$f\left(x_{i}|\prod ,\Theta \right)=\sum_{j=1}^{K}\pi_{\mathrm{ij}}\sum_{r=1}^{R}\eta_{\mathrm{ji}}G\left(x_{i}|\Theta_{\mathrm{ji}}\right)$$

where, G(x_i_|Θ_jr_) is the Gaussian distribution. Π = {π_ij_, j = 1, 2, …, K, i = 1, 2, …, N} is a parameter set, Θ = {μ_jr_, Σ_jr_, j = 1, 2, …, K, r = 1, 2, …, R} is a parameter set. π_ij_ represents the prior probability of the pixel x_i_ belonging to the j*th* category, η_jr_ represents the prior probability that this pixel point belongs to the r*th* Gaussian distribution in the j*th* category.

In order to increase noise immunity, Markov random field is introduced into the hierarchical Gaussian mixture model. The joint probability density function of Formula (19) is known by Bayes theorem of Formula (18).

(18) p(Π,Θ|Y)∝p(Y|Π,Θ)p(Π)

(19) $$p\left(\Pi \right)=Z^{-1}\exp\left\{\frac{1}{W}\sum_{i=1}^{N}\sum_{j=1}^{K}g_{\mathrm{ij}}^{\left(t\right)}{\log\pi }_{\mathrm{ij}}^{\left(t+1\right)}\right\}$$

(20) $$g_{\mathrm{ij}}^{\left(t\right)}=\exp\left[\frac{\beta }{{2N}_{i}}\sum_{m\in N_{i}}\left(z_{\mathrm{mj}}^{\left(t\right)}+\pi_{\mathrm{mj}}^{\left(t\right)}\right)\right]$$

where Π = {π_ij_, i = 1, 2, …, N, j = 1, 2, …, K} is parameter set, t is the iteration times, Z is the ormalized constant, the value of W is usually 1. g^(t)^_ij_ is the probability information that the pixel x_i_ belongs to the j*th* category combined with neighborhood information. β is the parameter of adjust the image smoothness. z_mj_ in Formula (21) is the posterior probability. N_i_ is the neighborhood information of pixel x_i_, the neighborhood is 3 × 3. ${\bar{N}}_{i}$ is the index value set of the neighborhood point of the pixel x_i_.

(21) $$Z_{\mathrm{ij}}^{\left(t\right)}=\pi_{\mathrm{ij}}^{\left(t\right)}G\left(X_{i}|\mu_{j}^{\left(t\right)},\sum_{j}^{\left(t\right)}\right)/\sum_{j=1}^{K}\pi_{\mathrm{ij}}^{\left(t\right)}G\left(X_{i}|\mu_{j}^{\left(t\right)},\sum_{j}^{\left(t\right)}\right)$$

During the iteration process, update μ_j_, $\sum_{j}$, π_ij_. The formulas are as follows,

(22) $$\mu_{j}^{t+1}=\sum_{i=1}^{N}z_{\mathrm{ij}}^{\left(t\right)}x_{i}/\sum_{i=1}^{N}z_{\mathrm{ij}}^{\left(t\right)}$$

(23) $$\sum_{j}^{t+1}={\left(Z_{\mathrm{ij}}^{\left(t\right)}\right)}^{T}\left(x_{i}-\mu_{j}^{t+1}\right)/\sum_{i=1}^{N}Z_{\mathrm{ij}}^{\left(t\right)}$$

(24) $$\pi_{\mathrm{ij}}^{\left(t+1\right)}=Z_{\mathrm{ij}}^{\left(t\right)}+G_{\mathrm{ij}}^{\left(t\right)}/\sum_{j=1}^{K}Z_{\mathrm{ij}}^{\left(t\right)}+G_{\mathrm{ij}}^{\left(t\right)}$$

Take the logarithm Likelihood function:

(25) $$\log^{\left(t\right)}=\sum_{i=1}^{N}\sum_{j=1}^{K}\left(Z_{\mathrm{ij}}^{\left(t\right)}\left({\log\pi }_{\mathrm{ij}}^{\left(t\right)}+\mathrm{logG}\left(x_{i}|\mu_{j}^{\left(t\right)},\sum_{j}^{\left(t\right)}\right)\right)\right)+\sum_{i=1}^{N}\sum_{j=1}^{K}g_{\mathrm{ij}}^{\left(t\right)}{\log\pi }_{\mathrm{ij}}^{\left(t\right)}$$

The iterative estimation is performed for the Formula (25). When log^(t+1)^−log^(t)^ < 0.1, then the iteration is stopped. The posterior probability function Z_ij_ is maximized, and then the image segmentation is realized.

## 3. Experiments

In this section, the performance of the proposed malignant melanoma image segmentation method is evaluated. At first, the experimental environment and the used dataset are described. Then, the results of our experiments are discussed.

### 3.1. Implementation Details

#### 3.1.1. Dataset and Training Settings

The experiments are conducted on a computer equipped with an NVIDIA GTX 3090 GPU. The manufacturer of the GPU is Shenzhen Colorful Technology Development Limited Corporation, and its country of origin is America. The memorycard is Kingston FURY 128 GB (32 G × 4) DDR4 3200. The motherboard is Gigabyte X570 AORUS PRO WIFI. Our method is implemented in CUDA 10.0, cuDNN 7.5, tensorflow2.0, and python 3.7. The malignant melanoma images are obtained partly from a well-known local hospital and from ISIC2018. Because the collected dataset is small, it is difficult to effectively train the neural network model. In this paper, the training data is the training set of ISIC2018 dataset, and the models are tested on ISIC2018 dataset and our collected dataset.

#### 3.1.2. Evaluation Metrics

For performance evaluation, we adopt several metrics recommended by the ISIC, namely, accuracy (Acc), specificity (SP), Jaccard index (JA), and Dice coefficient (Dice). The formulas for these evaluation criteria are:

(26) $$\mathrm{Acc}=\frac{\mathrm{TP}+\mathrm{TN}}{\mathrm{TP}+\mathrm{FN}+\mathrm{FP}+\mathrm{FN}}$$

(27) $$\mathrm{SP}=\frac{\mathrm{TN}}{\mathrm{TN}+\mathrm{FP}}$$

(28) $$\mathrm{JA}=\frac{\mathrm{TP}}{\mathrm{TP}+\mathrm{FN}+\mathrm{FP}}$$

(29) $$\mathrm{Dice}=\frac{2*\mathrm{TP}}{\mathrm{FP}+2*\mathrm{TP}+\mathrm{FN}}$$

where the skin lesion pixel is assumed to be a positive class and the background pixel is a negative class. TP, FP, TN, and FN represent the numbers of true positives, false positives, true negatives, and false negatives, respectively. Given a set of images, Acc is the accuracy, SP is the specificity. JA computes the similarity between the predicted region of the object present in the image and the ground truth region, representing the ratio of overlap between the predicted segmentation results and the ground truth labels. Dice is a common performance metric to evaluate the segmentation results, which measures the gap between the segmentation results and the label.

### 3.2. Experimental Results

#### 3.2.1. The Visualized Results after Applying the Neutrosophic Entropy

**Figure 8:**
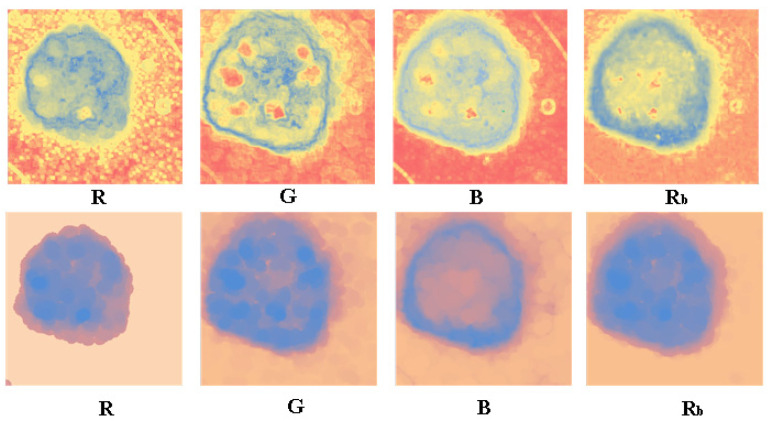
Results of neutrosophic entropy conversion. The first line is the unprocessed conversion results of each channel after the neutrosophic entropy conversion, and the second line is the conversion results of each channel with morphological processing after the neutrosophic entropy conversion.

In this paper, the results of R, G, B, and R_b_ are transformed by neutrosophic entropy, and the comparison figure is obtained through the three-color scale processing of blue-yellow-red as shown in Figure 8. The R component can effectively extract the edge information of the skin lesion area. However, only the inner edge information of the skin lesion is captured for the area band with a gradually shallower edge. At the same time, the characteristic influence of the lesion area is effectively suppressed. The background contains a lot of noise information caused by hair and bubbles in the dermoscopic image. The G component captures the edge information on the outside of the image, which weakens the influence of the background noise compared with the R component, but the influence on the inside of the lesion area is more significant. The B component also captures the outer edge features of the edge while effectively suppressing the influence of background noise. However, it is also affected by the feature of the lesion area inside the skin lesion. R_b_ combines the advantages between the two, and it can effectively divide the inner edge and the outer edge between the edge band and the blue-white veil background. At the same time, the influence of a large amount of background noise and internal features of skin lesions is suppressed. Finally, the morphology is used to remove a large amount of background noise, and at the same time, the small edge roughness is removed.

#### 3.2.2. The Visualized Results of the Clustering Methods

**Figure 9:**
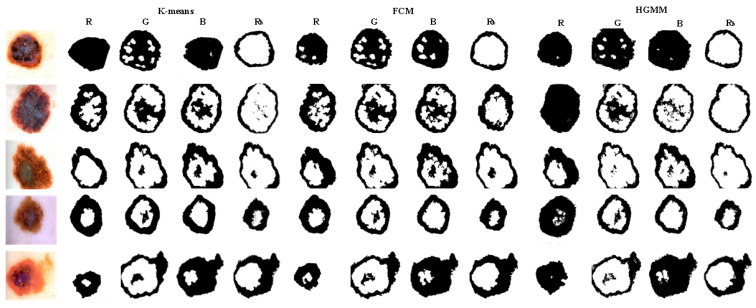
The segmentation results of different clustering methods.

The classic K-means, Fuzzy c-means (FCM), and Hierarchical Gaussian Mixture Model (HGMM) clustering methods are used to segment the images without morphological processing after the neutrosophic entropy processing, and the segmentation results are compared. As shown in Figure 9, the first to fourth columns show the clustering segmentation results of the R, G, B, and R_b_ components, respectively. As the result of experiment 3.2.1, the R_b_ component is superior to the single component in capturing the edge and denoising for the background and skin lesion features. The comparison results of the three clustering methods show that the HGMM algorithm can effectively capture the edge band features of the skin lesion. This method can maintain the structure boundary of the object region even if the structure edge of the image is blurred and the lines are irregular. On the other hand, the segmentation method based on neutrosophic entropy and the HGMM algorithm can effectively eliminate a large amount of noise in the background of the malignant melanoma image and the influence of lesion features of the skin lesion area.

#### 3.2.3. The Visualized Results of the Morphology Method

**Figure 10:**
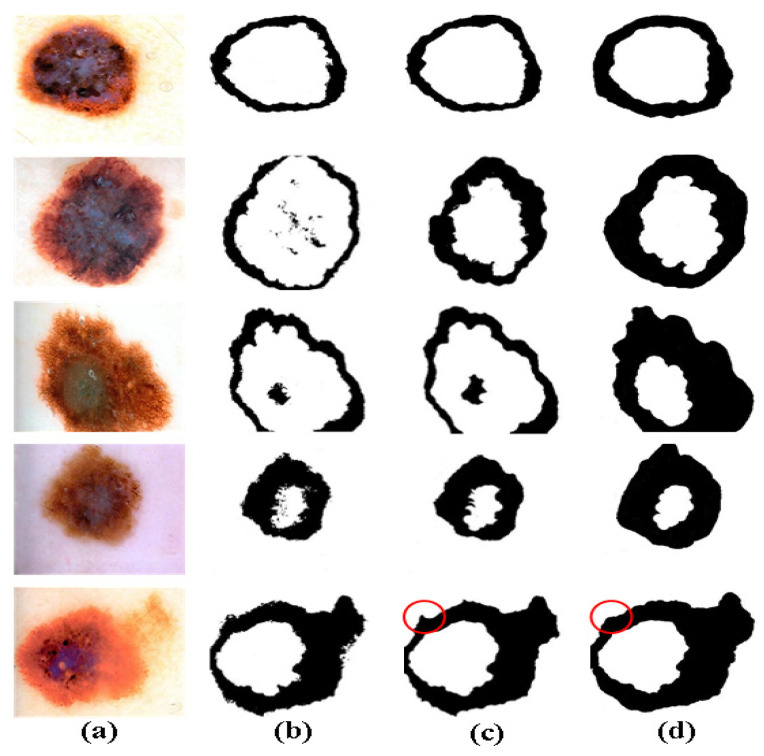
The results after adding morphology in different steps. (**a**) Raw malignant melanoma images (**b**) The results of the only R_b_ processing. (**c**) The results of firstly morphological processing followed by R_b_ processing. (**d**) The results of firstly R_b_ processing followed by morphological processing.

The combination of neutrosophic entropy and morphological technology is used to improve the identification and representation of edge band features in the lesion area of malignant melanoma images. The problem of irregular edges, blurring and color gradation of malignant melanoma images is well solved, and the skin lesion area segmentation performance is greatly improved. In the segmentation process of our method, some steps can add morphological methods to fill in holes and missing points. Since morphology will affect subsequent processing steps, the recall rate of image pixels is reduced. Therefore, the later is morphology added, the better the original information of the image can be guaranteed. However, at the same time, the irregular and frizzy edges of the skin lesion area will affect the accuracy of the malignant melanoma edge segmentation. Therefore, the results of adding morphological filling techniques in different links are compared to determine the best position of adding morphology. The visualization of the processing results after adding morphology in different steps is shown in Figure 10. It shows the transformation results after adding morphology of five malignant melanoma images in R_b_, first expansion and then R_b_, first R_b_ and then expansion.

It can be seen from Figure 10 that in the R_b_ component of the raw image, there is still noise even if channel R is subtracted from channel B due to a large amount of original noise in the image, which affects the final segmentation effect. A large amount of noise can be effectively avoided either by first conducting morphology and then extracting the R_b_ component or by extracting the R_b_ component and then conducting morphology. However, it can better capture the edge band information of the skin lesion area by first extracting the R_b_ component and then performing morphology for the internal features of the skin lesion area. At the same time, it has a better effect on edge burrs, edges, and corners, etc., as shown in the red circle in Figure 10. In summary, our method can improve segmentation accuracy through neutrosophic entropy conversion, and it can effectively remove noise by morphological methods. The improved image feature extraction method is helpful to capture irregular contour features effectively.

#### 3.2.4. Comparison with Other State-of-the-Art Segmentation Methods

The accurate segmentation technique for malignant melanoma proposed in this paper can be used in an unsupervised adaptive contour model. This technique helps to separate the required banded edge entities from the background and skin lesions. The image pixels are blurred by neutrosophic entropy, and then the information is collected from neighboring pixels of a similar class. Thus the effective segmentation of malignant melanoma of different intensities is ensured.

In order to prove the advantages of our method in fuzzy edge segmentation of malignant melanoma, our method is compared with other state-of-the-art segmentation methods. Our proposed algorithm achieved the highest score in most of the performance indicators used. In terms of evaluation indicators, Acc, SP, JA, and Dice are used. Table 1 provides a quantitative comparison between some state-of-the-art methods and our method. It can be seen from Table 1 that our method obtained the highest scores in Acc and SP, and it is only 0.002 worse than the optimal method in Dice. It shows that the segmentation result of our method is closer to the expected segmentation object. Meanwhile, compared with the deep learning method, our method greatly reduces the training time, and the results are relatively stable. Therefore, NeDSeM method in this paper has better segmentation performance than other advanced malignant melanoma segmentation methods.

#### 3.2.5. Visualization of the Results

**Figure 11:**
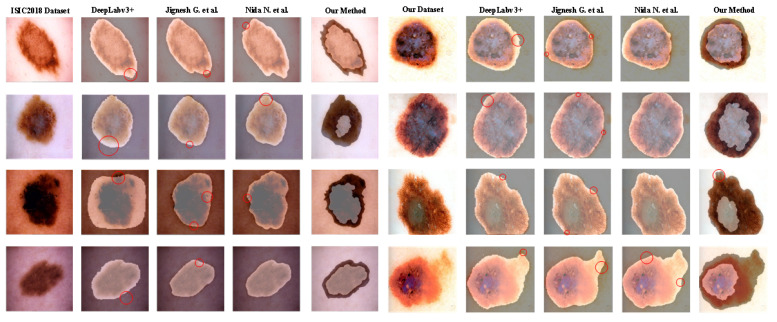
Visualization of the segmentation results of our dataset and ISIC2018 dataset.

To further evaluate our proposed method and visually evaluate the segmentation results of different methods, three baseline methods with high segmentation accuracy in Table 1 are selected. The segmentation results of eight representative malignant melanoma images from our dataset and the ISIC2018 dataset are shown in Figure 11. In the visual comparison results, the comparison results of the edge region quality after image segmentation with the different methods are showed. Combining the sample information and analyzing the lesion segmentation in Figure 11, it can be found that the contour of the lesion can be roughly segmented using Deeplabv3+, and a large amount of edge information is not effectively segmented. The contour of the skin lesion area can be successfully segmented by using Jignesh’s method. However, the result of segmentation still has the phenomenon of edge information loss. At the same time, many background skin pixels are inefficiently segmented, and the quality of the segmented edges is low. The edge information is effectively segmented and the segmentation result is smoother by using Nida’s method. However, there are still large deviations between the edge shape and the expected in the case of edge fuzzy is serious, which causes many skin pixels to be misclassified. In the segmentation results of our method, the segmentation edges are smoother, and the shape of the outer edges is more similar to the expected segmentation, which effectively captures the irregular edge shape features of the skin lesion area of a malignant melanoma image. However, the morphological method is used for smoothing, resulting in the situation of red circle marking in our method, that is, the violent and similar local edge fluctuations are not effectively segmented.

## 4. Discussion

Supervised segmentation methods have proved to be extremely effective for many problems in medical image segmentation, but the performance of these methods heavily depends on the quality of the labels. However, in practical applications, labels are usually lacking or even no labels, and large-scale labeling on medical images will also cost a lot of labor and time costs. Therefore, most of the current methods use unsupervised learning methods. Especially in recent years, with the further research of deep learning, unsupervised deep learning methods have made great progress in the field of image segmentation. They have great potential for effective image segmentation by utilizing large amounts of unlabeled data.

For semantic segmentation, some networks like U-Net also handle feature map reduction and detail loss. When recovering the resolution to the input size, the feature maps in the top and shallow layers are concatenated, and details are directly used to restore object boundaries. Although this skip connection mechanism can improve segmentation performance, directly taking features from shallow layers induces noise in the top layers, thus undermining the segmentation accuracy. Meanwhile, U-Net is more suitable for segmenting larger images, such as contours of brain region. However, malignant melanoma dermatoscopic images usually do not have such a large scale. Compared with the fixed human body structures in other medical images, the distribution of skin lesions in skin diseases is not very regular, and the semantics are more complex. So it is difficult to provide target object recognition information for U-Net in low resolution. This is the reason why the performance of U-Net-based method is lower than other methods in Table 1. Therefore, the characteristics of the U-Net method itself lead to scenes that are not suitable for dermoscopy image segmentation, and this paper also proves this point from the aspect of experiment. Similarly, in Deeplabv3+, since malignant melanoma dermoscopic images have no fixed rules similar to natural images, the distribution of medical features in the images is mostly irregular, with fuzzy and irregular edges. Therefore, the knowledge obtained from training is only included in the training set after Deeplabv3+ performs encoder and decoder operations. However, some novel edge shapes or pixel distribution features are difficult to be matched effectively by the model in verification and actual use. This results in the segmentation result of Deeplabv3+ in Figure 11, which divides a large amount of normal skin into skin lesion area. In the literature [27], image features are extracted by FPN, and Conditional Random Field (CRF) is used for fine segmentation of images. CRF obtains as much information as possible from neighboring points and encourages similar pixels to be assigned the same label, while pixels with a large difference are assigned different labels. In this way, CRF can segment the image at the boundary as much as possible, so as to obtain more accurate segmentation results. But CRF only operates on adjacent nodes, which will lose some contextual information. In response to this problem, the difference of the fully connected CRF model is that it describes the relationship between each pixel and all other pixels. The target region can be greatly refined and segmented on the image by using this model, which is also the main reason why the method in reference [27] in experiment 3.2.4 has better performance on the two indicators of JA and Dice. It is confirmed that the accuracy of the method in the literature [27] on the dermoscopy image segmentation task is higher than that of the FCN method and the SegNet method. However, this method consumes more computing power and is more expensive to restore the local detailed structure of the target.

Deep learning-based methods can automatically learn advanced features from raw data, however, this extraction method is not limited by human rules. In medicine, the impact of this situation will be magnified, even sometimes deviat from the strict requirements of the medical field and wast valuable experience accumulated over the years in the medical field. Therefore, we prefer human in rules, so that the final result has a certain degree of interpretability. Compared with the above deep learning methods, we focus on finding a good space or representation method to characterize the spatial features of pixels in malignant melanoma images. The complex dermoscopy image segmentation problem is transformed into a clustering problem in the neutrosophic space. The neutrosophic conversion and neutrosophic entropy methods proposed in this paper can flexibly map complex pixel distributions to different isentropic cylinders, enhance the accurate representation of the fuzziness and intuitiveness of pixel categories, and measure the similarity of the entropy values of all pixels in the space to achieve image segmentation. Experimental results show that the proposed method has a significant improvement in segmentation accuracy, and our method is better than the baseline methods on two metrics (ACC is 0.9678, SP is 0.9813). The visualization is validated on the real malignant melanoma dermoscopy image dataset collected by us in a well-known local hospital and ISIC2018 dataset, and the effectiveness of our method is also demonstrated through Experiment 3.2.4 and Experiment 3.2.5. In this paper, the banded segmentation edge is more in line with the needs of doctors for diagnosis, that is, en-bloc resection of the skin lesion area is guaranteed for different body incidence locations, which shows the effectiveness and practicability of the method in this paper.

## 5. Conclusions

Starting from the real application scenario, this paper simulates the real diagnosis process of the doctor through a long-term close cooperation with a well-known local hospital. Therefore, a representation of the fuzziness and intuitiveness in the doctor’s decision-making process is added into our proposed NeDSeM method. The fuzzy modeling of malignant melanoma images is realized by the neutrosophic conversion and neutrosophic entropy methods proposed in this paper. By segmenting the skin lesions of malignant melanoma images, it can assist doctors in the rapid and automatic preliminary diagnosis of dermoscopic images, unify the medical segmentation standards of dermoscopic images, reduce the influence of human bias, and reduce the heavy work pressure of doctors in diagnosing the skin lesion areas of a large number of dermatoscopic images. Our method mainly contains three modules, a NS domain conversion module, a NE domain conversion module, and an image segmentation module. In the NS domain conversion module, the gradient and closing operations in morphology are used to calculate indeterminacy (I) and true (T), respectively. The NS matrixes are obtained by the NS conversion method. The NE domain conversion module considers both the intuitiveness and the fuzziness of images and proposes a new neutrosophic entropy model to further improve the accuracy of the banded edges of the segmented images. The image segmentation module uses the morphological method to expand the NE matrixes, and the obtained DI matrixes are segmented by HGMM clustering method. The experimental evaluation of our malignant melanoma dataset and the ISIC2018 dataset demonstrates that our method has higher accuracy and better effect. In the future work, it will be verified that the NeDSeM method can be extended to other medical image segmentation tasks.

## Figures and Tables

**Table 1:** The comparison results of different segmentation methods on ISIC2018 dataset.

Method	Acc	SP	JA	Dice
U-net	0.8440	0.9255	0.7919	0.8066
DeepLabv3+	0.9352	0.9288	0.9009	0.8171
CDU-Net	0.8480	0.8560	0.8410	0.8132
Jignesh G. et al.	0.9592	0.970	0.8541	0.9152
Nida N. et al.	0.9400	0.9700	0.9100	0.9300
Our Method	0.9678	0.9813	0.8668	0.9281

## Data Availability

All data used in the experiments are from the local hospital. The datasets generated during the current study are available from the corresponding author on reasonable request.

## Supplementary Materials

The following supporting information can be downloaded at: https://www.mdpi.com/article/10.3390/e24060783/s1, File S1. This file contains the whole derivation process and properties of the single-valued neutrosophic entropy proposed in this paper.

## References

[1] Celebi M.E., Wen Q.U., Iyatomi H.I., Shimizu K.O., Zhou H., Schaefer G. (2015). A state-of-the-art survey on lesion border detection in dermoscopy images. Dermoscopy Image Anal..

[2] Efimenko M., Ignatev A., Koshechkin K. (2020). Review of medical image recognition technologies to detect melanomas using neural networks. BMC Bioinform..

[3] Wu H., Pan J., Li Z., Wen Z., Qin J. (2020). Automated skin lesion segmentation via an adaptive dual attention module. IEEE Trans. Med. Imaging.

[4] Xie F., Yang J., Liu J., Jiang Z., Zheng Y., Wang Y. (2020). Skin lesion segmentation using high-resolution convolutional neural network. Comput. Methods Programs Biomed..

[5] Nasiri S., Helsper J., Jung M., Fathi M. (2020). DePicT Melanoma Deep-CLASS: A deep convolutional neural networks approach to classify skin lesion images. BMC Bioinform..

[6] Pollastri F., Bolelli F., Paredes R., Grana C. (2020). Augmenting data with GANs to segment melanoma skin lesions. Multimed. Tools Appl..

[7] Zhou H., Schaefer G., Celebi M.E., Lin F., Liu T. (2011). Gradient vector flow with mean shift for skin lesion segmentation. Comput. Med. Imaging Graph..

[8] Shamsi M., Zoroofi R.A., Lucas C., Hasanabadi M.S., Alsharif M.R. (2008). Automatic facial skin segmentation based on em algorithm under varying illumination. IEICE Trans. Inf. Syst..

[9] Nadernejad E., Sharifzadeh S. (2013). A new method for image segmentation based on Fuzzy C-means algorithm on pixonal images formed by bilateral filtering. Signal Image Video Processing.

[10] Xu H., Mandal M. (2015). Epidermis segmentation in skin histopathological images based on thickness measurement and k-means algorithm. EURASIP J. Image Video Processing.

[11] Shi X., Li Y., Zhao Q. (2020). Flexible Hierarchical Gaussian Mixture Model for High-Resolution Remote Sensing Image Segmentation. Remote Sens..

[12] Wang Y., Chen Z., Xu Z. (2021). Brain MR image segmentation based on hierarchical Gaussian mixture model of MRF. J. Harbin Univ. Commer. (Nat. Sci. Ed.).

[13] Yu L., Chen H., Dou Q., Qin J., Heng P.A. (2016). Automated melanoma recognition in dermoscopy images via very deep residual networks. IEEE Trans. Med. Imaging.

[14] Oktay O., Schlemper J., Folgoc L.L., Lee M., Heinrich M., Misawa K., Mori K., McDonagh S., Hammerla N.Y., Kainz B. (2018). Attention u-net: Learning where to look for the pancreas. arXiv.

[15] Yuan Y., Chao M., Lo Y.C. (2017). Automatic skin lesion segmentation using deep fully convolutional networks with jaccard distance. IEEE Trans. Med. Imaging.

[16] Goodfellow I., Warde-Farley D., Mirza M., Courville A., Bengio Y. Maxout networks. Proceedings of the 30th International Conference on Machine Learning.

[17] Tang P., Liang Q., Yan X., Xiang S., Sun W., Zhang D., Coppola G. (2019). Efficient skin lesion segmentation using separable-Unet with stochastic weight averaging. Comput. Methods Programs Biomed..

[18] Huang G., Sun Y., Liu Z., Sedra D., Weinberger K.Q. (2016). Deep networks with stochastic depth. Proceedings of the European Conference on Computer Vision.

[19] Huang G., Liu Z., Van Der Maaten L., Weinberger K.Q. Densely connected convolutional networks. Proceedings of the IEEE Conference on Computer Vision and Pattern Recognition.

[20] Deshpande N.M., Gite S., Pradhan B., Kotecha K., Alamri A. (2022). Improved Otsu and Kapur approach for white blood cells segmentation based on LebTLBO optimization for the detection of Leukemia. Math. Biosci. Eng..

[21] Agrawal R., Kulkarni S., Walambe R., Deshpande M., Kotecha K. (2022). Deep dive in retinal fundus image segmentation using deep learning for retinopathy of prematurity. Multimed. Tools Appl..

[22] Gite S., Mishra A., Kotecha K. (2022). Enhanced lung image segmentation using deep learning. Neural Comput. Appl..

[23] Yu Z., Jiang X., Zhou F., Qin J., Ni D., Chen S., Lei B., Wang T. (2018). Melanoma recognition in dermoscopy images via aggregated deep convolutional features. IEEE Trans. Biomed. Eng..

[24] Chen L.C., Zhu Y., Papandreou G., Schroff F., Adam H. Encoder-decoder with atrous separable convolution for semantic image segmentation. Proceedings of the European Conference on Computer Vision (ECCV).

[25] Mu X., Pan H., Zhang K., Teng T., Bian X., Chen C. (2021). Channel Context and Dual-Domain Attention Based U-Net for Skin Lesion Attributes Segmentation. Proceedings of the International Conference of Pioneering Computer Scientists, Engineers and Educators.

[26] Chowdary G.J., Yathisha G.V. (2021). Exploring dual-attention mechanism with multi-scale feature extraction scheme for skin lesion segmentation. arXiv.

[27] Rehman H.U., Nida N., Shah S.A., Ahmad W., Faizi M.I., Anwar S.M. (2022). Automatic melanoma detection and segmentation in dermoscopy images using deep RetinaNet and conditional random fields. Multimed. Tools Appl..

[28] Wen J., Xuan S., Li Y., Peng Q., Gao Q. (2019). Image segmentation algorithm based on neutrosophic fuzzy clustering with non-local information. IET Image Processing.

[29] Song S., Jia Z., Yang J., Kasabov N.K. (2020). A Fast Image Segmentation Algorithm Based on Saliency Map and Neutrosophic Set Theory. IEEE Photonics J..

[30] Guo Y., Cheng H.D. (2009). New neutrosophic approach to image segmentation. Pattern Recognit..

[31] Cheng H.D., Guo Y. (2008). A new neutrosophic approach to image thresholding. New Math. Nat. Comput..

[32] Ashour A.S., Du C., Guo Y., Hawas A.R., Lai Y., Smarandache F. (2019). A Novel Neutrosophic Subsets Definition for Dermoscopic Image Segmentation. IEEE Access.

[33] Sert E., Alkan A. (2019). Image edge detection based on neutrosophic set approach combined with Chan–Vese algorithm. International J. Pattern Recognit. Artif. Intell..

[34] Zhang M., Zhang L., Cheng H.D. (2010). A neutrosophic approach to image segmentation based on watershed method. Signal Processing.

[35] Shan J., Cheng H.D., Wang Y. (2012). A novel segmentation method for breast ultrasound images based on neutrosophic l-means clustering. Med. Phys..

[36] Wang J., Zhang Q., Xu G. (2013). Extraction of operation characteristics in mechanical systems using genetic morphological filter. J. Vibroengineering.

[37] Shan C., Huang B., Li M. (2018). Binary morphological filtering of dominant scattering area residues for SAR target recognition. Comput. Intell. Neurosci..

[38] Özyurt F., Sert E., Avci E., Dogantekin E. (2019). Brain tumor detection based on Convolutional Neural Network with neutrosophic expert maximum fuzzy sure entropy. Measurement.

[39] Anter A.M., Hassenian A.E. (2019). CT liver tumor segmentation hybrid approach using neutrosophic sets, fast fuzzy c-means and adaptive watershed algorithm. Artif. Intell. Med..

[40] Wady S.H., Yousif R.Z., Hasan H.R. (2020). A Novel Intelligent System for Brain Tumor Diagnosis Based on a Composite Neutrosophic-Slantlet Transform Domain for Statistical Texture Feature Extraction. BioMed Res. Int..

[41] Majumdar P., Samanta S.K. (2014). On similarity and entropy of neutrosophic sets. J. Intell. Fuzzy Syst..

[42] Aydoğdu A. (2015). On similarity and entropy of single valued neutrosophic sets. Gen. Math. Notes.

