# Supplementary material for: NeDSeM: Neutrosophy Domain-Based Segmentation Method for Malignant Melanoma Images
Source: Entropy (Basel). 2022 Jun 2;24(6):783. doi: 10.3390/e24060783 (PMC9222744; doi:10.3390/e24060783)
Supplement: Supplementary file 1 [file entropy-24-00783-s001.zip › entropy-1711206-supplementary.pdf]

Detailed derivation process of single-valued neutrosophic entropy

A.1. The single-valued neutrosophic number

Definition 1: Suppose X is a domain. In this paper, any malignant melanoma image is a domain. $A=\{x, <T_A(x),I_A(x),F_A(x)>|x\in X\}$ is a single-valued neutrosophic set, where $T_A(x)$, $I_A(x)$ and $F_A(x)$ are the membership degree, the hesitancy degree and the non-membership degree of the element x on A, respectively. For $\forall x\in X$, have $T_A(x)\in[0,1]$, $I_A(x)\in[0,1]$, $F_A(x)\in[0,1]$, and $0\le T_A(x)+I_A(x)+F_A(x)\le 3$. For convenience of study, it is called $\alpha=<T, I, F>$ is a single-valued neutrosophic number.

Definition 2: Suppose two single-valued neutrosophic numbers $\alpha_1=<T_1, I_1, F_1>$ and $\alpha_2=<T_2, I_2, F_2>$. If $T_1<T_2$, $I_1>I_2$ and $F_1>F_2$, then called $\alpha_1<\alpha_2$.

Definition 3: Suppose two single-valued neutrosophic numbers $\alpha_1=<T_1, I_1, F_1>$ and $\alpha_2=<T_2, I_2, F_2>$, then

(1) $\alpha_1\cup\alpha_2=<\max(T_1,T_2), \min(I_1,I_2), \min(F_1,F_2)>$,

(2) $\alpha_1\cap\alpha_2=<\min(T_1,T_2), \max(I_1,I_2), \max(F_1,F_2)>$.

Definition 4: Suppose $\alpha=<T, I, F>$ is a single-valued neutrosophic number, then the complement set of α is $\alpha^c=<F, 1-I, T>$. And satisfied $(\alpha^c)^c=\alpha$.

Definition 5: Suppose two single-valued neutrosophic numbers $\alpha_1=<T_1, I_1, F_1>$ and $\alpha_2=<T_2, I_2, F_2>$, the constant $k>0$, then

(1) $\alpha_1+\alpha_2= <T_1+T_2-T_1T_2, I_1I_2, F_1F_2>$;

(2) $\alpha_1\times\alpha_2= <T_1T_2, I_1+I_2-I_1I_2, F_1+F_2-F_1F_2>$;

(3) $k\alpha_1= <1-(1-T_1)^k, (I_1)^k, (F_1)^k>$;

(4) $(\alpha_1)^k= <(T_1)^k, 1-(1-I_1)^k, 1-(1-F_1)^k>$.

A.2. The existing single-valued neutrosophic entropy measure

P.Majumdar and S. K. Samanta (2014) proposed the axiomatic definition of the single-valued neutrosophic entropy on the basis of intuitionistic fuzzy entropy to measure the degree of uncertainty of single-valued neutrosophic set.

Mapping E: $SVNS(X)\to[0,1]$ is called single-valued neutrosophic entropy, and the following conditions must be met:

(1) $E(A)=0$. If and only if A is a crisp set;

(2) $E(A)=1$. If and only if $<t_A(x_i), i_A(x_i), f_A(x_i)>=<0.5, 0.5, 0.5>$, $\forall x_i\in X$;

(3) $E(A)\ge E(B)$. If A is more uncertain than B, that is $t_A(x_i)+f_A(x_i)\le t_B(x_i)+f_B(x_i)$, and $|i_A(x_i)-i_A^c(x_i)|\le|i_B(x_i)-i_B^c(x_i)|$.

(4) $E(A)=E(A^c)$.

Then, Ali proposed a single-valued neutrosophic entropy E:

$$E=\frac{1}{n}\sum_{i=1}^{n}(1-\frac{1}{b-a}\int_{a}^{b}|t-f||i-i_c|dx)$$

The neutrosophic set contains both the membership information, namely t, f, and the unknown information, namely i. Therefore, the neutrosophic set should be composed of two parts, one is fuzziness, which can be measured by $|t-f|$, and the other is intuitiveness, which can be measured by $|i-i_c|$.

A.3. The axiomatic definition and geometric interpretation of the existing single-valued neutrosophic entropy

Definition 6: (The axiomatic definition of single-valued neutrosophic entropy)

If the mapping E: SVNS(X)→[0,1] is a single-valued neutrosophic entropy, and the following conditions must be met:

(1) E(A)=0. If and only if A is a crisp set;

(2) E(A)=1. If and only if $t_A(x_i)=f_A(x_i)$, for any $x_i\in X$, $i_A(x_i)=0.5$;

(3) When $|i_A(x_i)-i_A{}^c(x_i)|=|i_B(x_i)-i_B{}^c(x_i)|$, that is $i_A(x_i)=i_B(x_i)$ or $i_A(x_i)=1-i_B(x_i)$, and $|t_A(x_i)-f_A(x_i)|<|t_B(x_i)-f_B(x_i)|$, for any $x_i\in X$, then has E(A)>E(B);

(4) When $|t_A(x_i)-f_A(x_i)|=|t_B(x_i)-f_B(x_i)|$and $|i_A(x_i)-i_A{}^c(x_i)|=|i_B(x_i)-i_B{}^c(x_i)|$,

that is,

$0.5<i_A(x_i)<i_B(x_i)$

or

$i_B(x_i)<i_A(x_i)<0.5$

or

$i_A(x_i)<1-i_B(x_i)$ and $i_B<0.5<i_A$

or

$i_B(x_i)>1-i_A(x_i)$ and $i_A<0.5<i_B$. For any $x_i\in X$, then has E(A)>E(B);

(5) $E(A)=E(A^c)$;

(6) With the change of the single-valued neutrosophic set, the value of single-valued neutrosophic entropy is continuous on [0,1].

The above axiomatic definition is explained in the geometric meaning:

Suppose $A=\{x, <T_A(x),I_A(x),F_A(x)>|x\in X\}$ is a single-valued neutrosophic set, where $T_A(x)$ and $F_A(x)$ are the membership degree and non-membership degree of the element x on A, respectively, and $I_A(x)$ is the hesitancy degree of the element x on A. Now the rectangular coordinate system is

established by the membership degree axis (X axis), the non-membership degree axis (Y axis), and the hesitancy degree axis (Z axis), respectively. So that <$t_A(x)$,$i_A(x)$,$f_A(x)$> corresponding to each x can has its corresponding point in the three-dimensional rectangular coordinate system, that is, point x($t_A(x)$,$f_A(x)$,$i_A(x)$). Since $0 \le t_A(x) \le 1$, $0 \le f_A(x) \le 1$, $0 \le i_A(x) \le 1$, and their sum is $0 \le t+f+i \le 3$, it can be seen that any x corresponds to a point in a cube with an intercept of 1. As shown in Fig.A.1, A(1,0,0), C(0,1,0), and E(0,0,1) are the points corresponding to the intercept of 1 on the three coordinate axes in the plane, and B(1,1,0), D(0,1,1), F(1,0,1), O(0,0,0), and G(1,1,1) are the remaining vertices in the cube. According to the axiomatic definition, the entropy corresponding to $\forall x_i$ point in the cube should satisfy $0 \le E(x_i) \le 1$, and E(A)=E(B)=E(C)=E(D)=E(E)=E(F)=E(G)=E(O)=0.

Obviously, all points on plane EGBO have membership degree equal to non-membership degree, that is, $|t-f|=0$, and the fuzziness is strongest at this time. As the points on plane OABC move toward plane FEDG, obviously, the hesitancy degree is increasing. However, the fuzziness of hesitancy degree increases first and then decreases. That is, the value of $|i-i_c|$ changes from 1 to 0 and then to 1 and reaches the minimum at i=0.5 and $|i-i_c|=0$. That is, the fuzziness of hesitancy degree of all points on the plane z=0.5 is the strongest. All points on the intersection line between plane FEDG and plane z=0.5 have maximum fuzziness and intuitiveness, that is, all points on this line have maximum entropy, E=1. The intuitiveness of all points in the cube is symmetric about plane z=0.5, and the fuzziness is symmetric about plane FEDG.

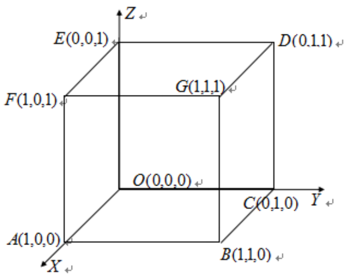

Fig.A.1. The spatial graph of single-valued neutrosophic entropy.

In the cube, some planes are made parallel to plane EGOB. As shown in Fig.A.2, each plane corresponds to a unique difference value $|t-f|$ between membership degree and non-membership degree. That is to say, the difference value $|t-f|$ of the points on each plane is the same, and they have the same fuzziness, and the values of $|t-f|$ on both sides of plane EGOB are symmetric about it. So we can only study the intersection line of these planes with plane FACD. As shown in Fig.A.3, suppose points x_1(0.5,0.1,0.4), x_2(0.1,0.3,0.2), x_3(0.3,0.5,0.5) and x_4(0.2,0.6,0.4), and their corresponding neutrosophic numbers are <0.5,0.4,0.1>, <0.1,0.2,0.3>, <0.3,0.5,0.5>and <0.2,0.4,0.6>, respectively. The coordinate of the point transformed from (x,y,z) to plane FACD is $(\frac{1+x-y}{2},\frac{1-x+y}{2},z)$, so there are transformed points x_1(0.7,0.3,0.4), x_2(0.4,0.6,0.2), x_3(0.4,0.6,0.5) and x_4(0.3,0.7,0.4). It can be seen that on line segment $|t-f|=0.2$, x_3 is closer to the midpoint of line segment than x_2, so $E(x_3)>E(x_2)$. Point x_1 and point x_4 are symmetric about $|t-f|=0$, so $E(x_1)=E(x_4)$.

Similarly, some planes are made parallel to plane ABCO in the cube, as shown in Fig.A.4. Each plane corresponds to a unique hesitancy degree value i, which obviously corresponds to a unique value of $|i-i_c|$. That is to say, the difference value $|i-i_c|$ of the points on each plane is the same, and they have the same intuitiveness, and the values of $|i-i_c|$ on both sides of plane z=0.5 are symmetric about it. So we can only study the intersection line of these planes with plane FACD. As shown in Fig.A.5, suppose points $x_5(0.2,0.2,0.7)$, $x_6(0.4,0.2,0.4)$, $x_7(0.6,0.7,0.4)$ and $x_8(0.7,0.7,0.3)$, and their corresponding neutrosophic numbers are <0.2,0.7,0.2>, <0.4,0.4,0.2>, <0.6,0.4,0.7> and <0.7,0.3,0.7>, respectively. The coordinate of the point transformed from (x,y,z) to plane FACD is $(\frac{1+x-y}{2},\frac{1-x+y}{2},z)$, so there are transformed points $x_1(0.5,0.5,0.7)$, $x_2(0.6,0.4,0.4)$, $x_3(0.45,0.55,0.4)$ and $x_4(0.5,0.5,0.3)$. It can be seen that on line segment $|i-i_c|=0.2$, x_7 is closer to the midpoint of the line segment than x_6, so $E(x_7)>E(x_6)$. Point x_5 and point x_8 are symmetric about $|i-i_c|=0$, so $E(x_5)>E(x_8)$.

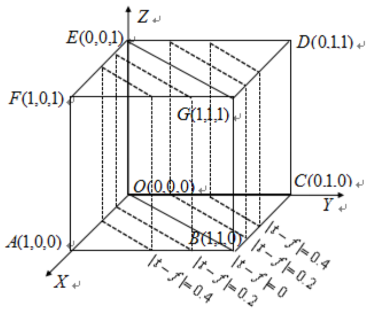

Fig.A.2. Isosurface |t−f| in the cube of single-valued neutrosophic entropy.

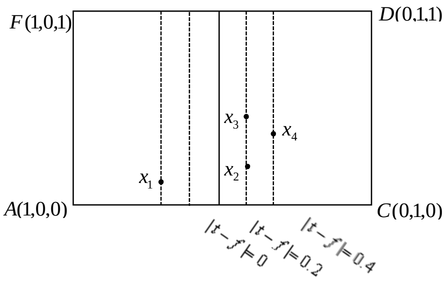

Fig.A.3. The intersection of the isosurface |t−f| and the two-dimensional plane ACDF(projection).

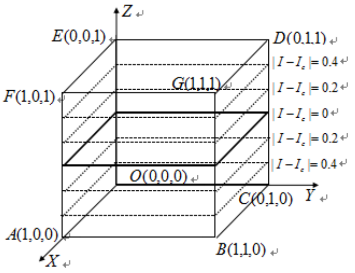

Fig.A.4. Isosurface $|i-i_c|$ in the cube of single-valued neutrosophic entropy.

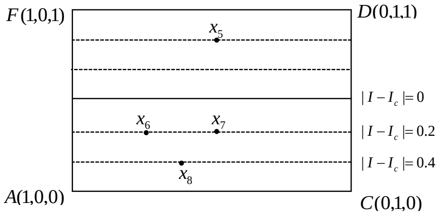

Fig.A.5. The intersection of the isosurface $|i-i_c|$ and the two-dimensional plane ACDF(projection).

Define intuitiveness $|I-I_c|=|2I-1|$ and fuzziness $|T-T_c||F-F_c|=(T-F)^2$, where $I_c=1-I$, $T_c=F$ and $F_c=T$. Then when $|I-I_c|=0$, that is, $I=I_c=0.5$ and $|T-F|=0$, the entropy is considered to be the maximum. The straight line $\begin{cases} I=0.5 \\ T=F \end{cases}$ is made as the axis and d as the radius to make the cylindrical surface. In the process of moving from point A to point B on the surface, the intuitiveness is decreasing, and the fuzziness is increasing. The uncertainty remains unchanged, that is, the entropy is the same. Point A and point B inside the cube are on the same curved surface.

A.4. The proposed single-valued neutrosophic entropy

Now the comparison of entropy between a point x_i in the cube and several key points around it is considered, as shown in Fig.A.6(a) and Fig.A.6(b). For the convenience of comparison, all points are projected on plane ACDF, as shown in Fig.A.7. Finally, only the entropy between Q_i and x^*_i is compared, and it has $E(Q_4)<E(x_i)<E(Q_5)$, $E(Q_7)<E(x_i)<E(Q_2)$. Because of $E(x_i)<E(Q_2)$ and $E(Q_2)<E(Q_3)$, $E(x_i)<E(Q_3)$ is obtained. The same is true for $E(x_i)>E(Q_6)$, that is, $E(Q_6)<E(x_i)<E(Q_3)$. However, it is impossible to compare point x_i with point Q_1 and point Q_8, because when x_i moves to Q_1 or Q_8, increase-decrease characteristics of its intuitiveness and fuzziness are opposite. It is impossible to intuitively judge which aspect is more important through geometric figures, so the entropy cannot be compared. To solve this problem, a cylindrical surface is made with the intersection line $\begin{cases} z=0.5 \\ t=f \end{cases}$ of plane z=0.5 and plane EGBO as the axis and d as the radius, as shown in Fig.A.8. As point A approaches point B, the fuzziness increases and the intuitiveness decreases. We can assume that their interactions cancel each other out, so that the uncertainty information at this point is constant, that is, the entropy is constant, that is, E(A)=E(B), this arc is called an isentropic cylinder.

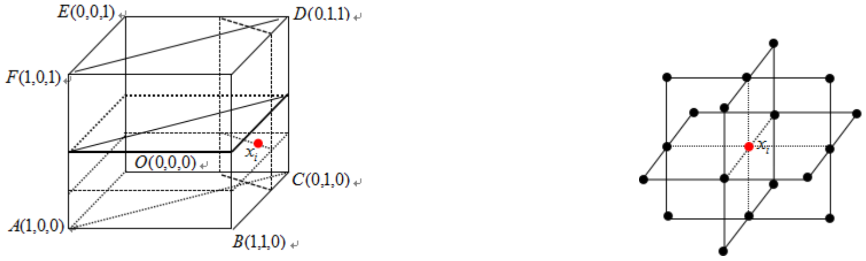

(a) (b)

Fig.A.6. (a) The position of point x_i in the cube (b) The key points around point x_i.

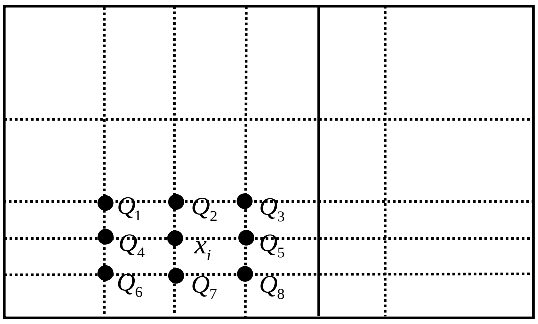

Fig.A.7. The position relationship between point x_i and surrounding key points projected on plane ACDF.

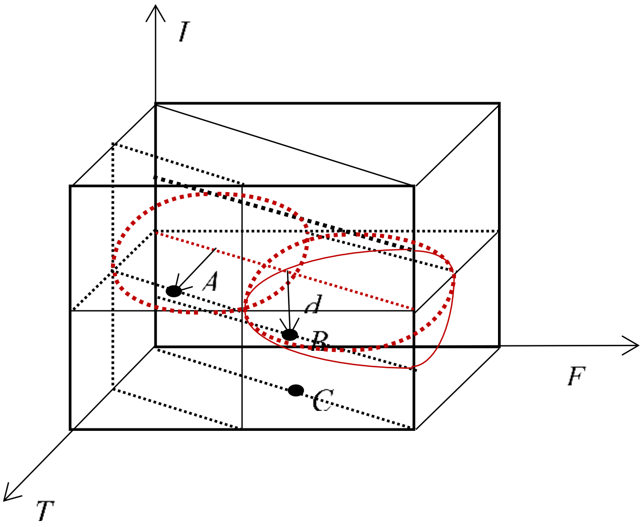

Fig.A.8. The geometric meaning of single-valued neutrosophic entropy.

As can be seen from Fig.A.8, the points on the isentropic cylinder have the feature of equal entropy, and the entropy has an obvious decreasing trend with the increase of d. Therefore, the neutrosophic entropy corresponding to this point can be measured according to the distance between any point in the cube and the straight line $\begin{cases} z = 0.5 \\ t = f \end{cases}$, A more reasonable and reliable entropy formula can be established based on this. Theorem 1 can be obtained after the research.

Theorem 1: Suppose domains X={$x_1,x_2,\ldots,x_n$}, A={x_i,<$t_A(x_i)$,$i_A(x_i)$, $f_A(x_i)$>|$x_i \in X$}$\in$SVNS(X), if

$$E(A) = \frac{1}{n}\sum_{i=1}^{n}\left\{1 - \frac{2\sqrt{3}}{3}\sqrt{\frac{(t_A(x_i) - f_A(x_i))^2}{2} + (\frac{|I_A(x_i) - I_{A^c}(x_i)|}{2})^2}\right\}$$

$$= \frac{1}{n}\sum_{i=1}^{n}\left\{1 - \frac{2\sqrt{3}}{3}\sqrt{\frac{(t_A(x_i) - f_A(x_i))^2}{2} + (\frac{|1 - 2I_A(x_i)|}{2})^2}\right\}$$

Then E is the entropy of the intuitionistic fuzzy set.

Proof: It is now proved that entropy E satisfies all the conditions of axiomatic definition.

(1) E(A)=0. If and only if

$$1-\frac{2\sqrt{3}}{3}\sqrt{\frac{(t_A(x_i)-f_A(x_i))^2}{2}+(\frac{|1-2i_A(x_i)|}{2})^2}=0,$$

$$\sqrt{\frac{(t_A(x_i)-f_A(x_i))^2}{2}+\frac{(1-2i_A(x_i))^2}{4}}=\frac{\sqrt{3}}{2},$$

$$2(t_A(x_i)-f_A(x_i))^2+(1-2i_A(x_i))^2=3.$$

That is, for any $x_i \in X$, has $t_A(x_i)=1$, $f_A(x_i)=0$, $i_A(x_i)=0$, or $t_A(x_i)=0$, $f_A(x_i)=1$, $i_A(x_i)=0$ or $t_A(x_i)=1$, $f_A(x_i)=0$, $i_A(x_i)=1$ or $t_A(x_i)=0$, $f_A(x_i)=1$, $i_A(x_i)=1$. So A is a crisp set;

(2) E(A)=1. If and only if

$$1-\frac{2\sqrt{3}}{3}\sqrt{\frac{(t_A(x_i)-f_A(x_i))^2}{2}+(\frac{|1-2i_A(x_i)|}{2})^2}=1,$$

$$\sqrt{\frac{(t_A(x_i)-f_A(x_i))^2}{2}+\frac{(1-2i_A(x_i))^2}{4}}=0.$$

That is, for any $x_i \in X$, has $t_A(x_i)=f_A(x_i)$, $i_A(x_i)=0.5$.

(3) i. When $i_A(x_i)=i_B(x_i)=\alpha$, $|t_A(x_i)-f_A(x_i)|=a$, $|t_B(x_i)-f_B(x_i)|=b$ and $a<b$,

For any $x_i \in X$,

$$\begin{aligned}&E(A)-E(B)\\&=\left(1-\frac{2\sqrt{3}}{3}\sqrt{\frac{(t_A(x_i)-f_A(x_i))^2}{2}+(\frac{|1-2i_A(x_i)|}{2})^2}\right)-\left(1-\frac{2\sqrt{3}}{3}\sqrt{\frac{(t_B(x_i)-f_B(x_i))^2}{2}+(\frac{|1-2i_B(x_i)|}{2})^2}\right)\\&=\left(1-\frac{2\sqrt{3}}{3}\sqrt{\frac{a^2}{2}+\frac{(1-2\alpha)^2}{4}}\right)-\left(1-\frac{2\sqrt{3}}{3}\sqrt{\frac{b^2}{2}+\frac{(1-2\alpha)^2}{4}}\right)\\&=\left(\frac{2\sqrt{3}}{3}\left(\sqrt{\frac{b^2}{2}+\frac{(1-2\alpha)^2}{4}}-\sqrt{\frac{a^2}{2}+\frac{(1-2\alpha)^2}{4}}\right)\right)\end{aligned}$$

Because of $0<a<b<1$, so $\frac{a^2}{2}<\frac{b^2}{2}$, $\frac{a^2}{2}+\frac{(1-2\alpha)^2}{4}<\frac{b^2}{2}+\frac{(1-2\alpha)^2}{4}$,

$\sqrt{\frac{a^2}{2}+\frac{(1-2\alpha)^2}{4}}<\sqrt{\frac{b^2}{2}+\frac{(1-2\alpha)^2}{4}}$, so has $\sqrt{\frac{b^2}{2}+(1-\alpha)^2}-\sqrt{\frac{a^2}{2}+(1-\alpha)^2}>0$, then E(A)>E(B) is established.

ii. When $i_A(x_i)=1-i_B(x_i)=\alpha$, $|t_A(x_i)-f_A(x_i)|=a$, $|t_B(x_i)-f_B(x_i)|=b$ and $a<b$,

For any $x_i \in X$,

$$E(A)-E(B)$$

$$=\left(1-\frac{2\sqrt{3}}{3}\sqrt{\frac{(t_A(x_i)-f_A(x_i))^2}{2}+(\frac{|1-2i_A(x_i)|}{2})^2}\right)-\left(1-\frac{2\sqrt{3}}{3}\sqrt{\frac{(t_B(x_i)-f_B(x_i))^2}{2}+(\frac{|1-2i_B(x_i)|}{2})^2}\right)$$

$$=\left(1-\frac{2\sqrt{3}}{3}\sqrt{\frac{a^2}{2}+\frac{(1-2\alpha)^2}{4}}\right)-\left(1-\frac{2\sqrt{3}}{3}\sqrt{\frac{b^2}{2}+\frac{(2\alpha-1)^2}{4}}\right)$$

$$=\left(\frac{2\sqrt{3}}{3}\left(\sqrt{\frac{b^2}{2}+\frac{(2\alpha-1)^2}{4}}-\sqrt{\frac{a^2}{2}+\frac{(1-2\alpha)^2}{4}}\right)\right)$$

Because of 0<a<b<1, so $\frac{a^2}{2}<\frac{b^2}{2}$, $\frac{a^2}{2}+\frac{(1-2\alpha)^2}{4}<\frac{b^2}{2}+\frac{(1-2\alpha)^2}{4}$,

$\sqrt{\frac{a^2}{2}+\frac{(1-2\alpha)^2}{4}}<\sqrt{\frac{b^2}{2}+\frac{(1-2\alpha)^2}{4}}$, so has $\sqrt{\frac{b^2}{2}+(1-\alpha)^2}-\sqrt{\frac{a^2}{2}+(1-\alpha)^2}>0$, then E(A)>E(B) is established.

To sum up, E(A)>E(B) is established.

(4) Let $i_A(x_i)=\alpha$, $i_B(x_i)=\beta$, $|t_A(x_i)-f_A(x_i)|=|t_B(x_i)-f_B(x_i)|=b$,

For any $x_i\in X$,

$$E(A)-E(B)$$

$$=\left(1-\frac{2\sqrt{3}}{3}\sqrt{\frac{(t_A(x_i)-f_A(x_i))^2}{2}+(\frac{|1-2i_A(x_i)|}{2})^2}\right)-\left(1-\frac{2\sqrt{3}}{3}\sqrt{\frac{(t_B(x_i)-f_B(x_i))^2}{2}+(\frac{|1-2i_B(x_i)|}{2})^2}\right)$$

$$=\left(1-\frac{2\sqrt{3}}{3}\sqrt{\frac{a^2}{2}+\frac{(2\alpha-1)^2}{4}}\right)-\left(1-\frac{2\sqrt{3}}{3}\sqrt{\frac{b^2}{2}+\frac{(2\beta-1)^2}{4}}\right)$$

$$=\left(\frac{2\sqrt{3}}{3}\left(\sqrt{\frac{b^2}{2}+\frac{(2\beta-1)^2}{4}}-\sqrt{\frac{b^2}{2}+\frac{(2\alpha-1)^2}{4}}\right)\right)$$

i. If 0.5<α<β,

Because of 0.5<α<β, so 0<2α–1<2β–1, $(2\alpha-1)^2<(2\beta-1)^2$,

$\sqrt{\frac{b^2}{2}+\frac{(2\beta-1)^2}{4}}>\sqrt{\frac{b^2}{2}+\frac{(2\alpha-1)^2}{4}}$, so has $\sqrt{\frac{b^2}{2}+\frac{(2\beta-1)^2}{4}}-\sqrt{\frac{b^2}{2}+\frac{(2\alpha-1)^2}{4}}>0$, then E(A)>E(B) is established.

ii. If β<α<0.5,

Because of β<α<0.5,so 0<1–2α<1–2β, $(1-2\alpha)^2<(1-2\beta)^2$,

$\sqrt{\frac{b^2}{2}+\frac{(1-2\beta)^2}{4}}>\sqrt{\frac{b^2}{2}+\frac{(1-2\alpha)^2}{4}}$, so has $\sqrt{\frac{b^2}{2}+\frac{(1-2\beta)^2}{4}}-\sqrt{\frac{b^2}{2}+\frac{(1-2\alpha)^2}{4}}>0$, then E(A)>E(B) is established.

iii. If α<1–β and β<0.5<α,

Because of $\alpha<1-\beta$ and $\beta<0.5<\alpha$, so $2\alpha-1<1-2\beta$, $(2\alpha-1)^2<(1-2\beta)^2$,

$\sqrt{\frac{b^2}{2}+\frac{(1-2\beta)^2}{4}} > \sqrt{\frac{b^2}{2}+\frac{(1-2\alpha)^2}{4}}$, so has $\sqrt{\frac{b^2}{2}+\frac{(1-2\beta)^2}{4}} - \sqrt{\frac{b^2}{2}+\frac{(1-2\alpha)^2}{4}} > 0$, then $E(A)>E(B)$ is established.

iv. If $\beta>1-\alpha$ and $\alpha<0.5<\beta$,

Because of $\beta>1-\alpha$ and $\alpha<0.5<\beta$, so $1-2\alpha<2\beta-1$, $(1-2\alpha)^2<(2\beta-1)^2$,

$\sqrt{\frac{b^2}{2}+\frac{(2\beta-1)^2}{4}} > \sqrt{\frac{b^2}{2}+\frac{(1-2\alpha)^2}{4}}$, so has $\sqrt{\frac{b^2}{2}+\frac{(2\beta-1)^2}{4}} - \sqrt{\frac{b^2}{2}+\frac{(1-2\alpha)^2}{4}} > 0$, then $E(A)>E(B)$ is established.

To sum up, $E(A)>E(B)$ is established.

(5) Suppose $A=\{x_i, <t_A(x_i),i_A(x_i),f_A(x_i)>|x_i\in X\}$,

Then $A^c=\{x_i, <t_A{}^c(x_i),i_A{}^c(x_i),f_A{}^c(x_i)>|x_i\in X\}$, where $t_A{}^c(x_i)=f_A(x_i)$, $f_A{}^c(x_i)=t_A(x_i)$, $f_{A^c}(x_i)=t_A(x_i)$.

Because of

$$E(A)=1-\frac{2\sqrt{3}}{3}\sqrt{\frac{(t_A(x_i)-f_A(x_i))^2}{2}+\frac{|i_A(x_i)-i_{A^c}(x_i)|^2}{4}},$$

$$\begin{aligned}E(A^c)&=1-\frac{2\sqrt{3}}{3}\sqrt{\frac{(t_{A^c}(x_i)-f_{A^c}(x_i))^2}{2}+\frac{|i_{A^c}(x_i)-i_A(x_i)|^2}{4}}\\&=1-\frac{2\sqrt{3}}{3}\sqrt{\frac{(f_A(x_i)-t_A(x_i))^2}{2}+\frac{|i_{A^c}(x_i)-i_A(x_i)|^2}{4}}\end{aligned},$$

Then $E(A)=E(A^c)$.

(6) It is obvious that the single-valued neutrosophic entropy E(A) continuously changes on [0,1] with different intuitionistic fuzzy sets.
